# A Case Series on Acenocoumarol and Linezolid Drug Interaction

**DOI:** 10.7759/cureus.52753

**Published:** 2024-01-22

**Authors:** Indrani Sarma, Narendra Sharma, Bhabananda Das, Jyoti Kalita

**Affiliations:** 1 Pharmacology, All India Institute of Medical Sciences, Guwahati, Guwahati, IND; 2 Anaesthesiology, Health City Hospital, Guwahati, IND; 3 Cardiothoracic and Vascular Surgery, Health City Hospital, Guwahati, IND

**Keywords:** raised inr, linezolid, drug, interaction, acenocoumarol

## Abstract

Acenocoumarol is one of the most common drugs used as a part of the management of patients undergoing cardiac surgery. Linezolid, on the other hand, is an antibiotic prescribed post-operatively. Reports of any interaction between the two are very few. Here, we are presenting four case reports of patients admitted to the Cardio Thoracic and Vascular Surgery Department of a tertiary healthcare center in North East India. Drug-drug interactions can lead to long-term and life-threatening effects, and also hamper the management of patients post-operatively. Due to the limited literature, assessing such interactions is difficult. The cases reported here were treated with fresh frozen plasma, and the patients responded well to the treatment and were discharged without further complications.

## Introduction

Acenocoumarol is primarily used for the prevention of deep vein thrombosis, and it has other off-label uses, such as in stroke patients with low ventricular ejection fraction [1]. Anti-platelet and anticoagulant therapies are crucial parts of the management of patients undergoing cardiac surgery. Vitamin K antagonists (VKAs), such as warfarin, are frequently used after mechanical heart valve implantation or in cases of atrial fibrillation. Bleeding is a commonly associated adverse event. These limitations of VKAs have led to the development of new oral anticoagulants, as found by Head SJ et al. [2]. Compared to warfarin, acenocoumarol has advantages such as a faster onset of action, less enzymatic metabolism by CYP2C9, more stable anticoagulation, and better reversal of anticoagulant action [3]. In India, acenocoumarol (Acitrom) is a popular choice for oral anticoagulant therapy in major cardiac surgeries [4]. VKAs have a narrow therapeutic index, requiring frequent dose adjustments [5]. Ensuring their safety involves closely monitoring prothrombin time, expressed as the International Normalised Ratio (INR). However, this frequent monitoring can be expensive and cumbersome for patients.

Linezolid is an antibiotic used to treat infections caused by Gram-positive bacteria resistant to other antibiotics, such as streptococci, vancomycin-resistant enterococci (VRE), and methicillin-resistant Staphylococcus aureus (MRSA) [6,7]. Its main uses are for skin infections and pneumonia [7][8]. It is often the first-line postoperative antibiotic in cardiac surgeries. While linezolid is relatively safe when used for short periods, longer use may cause nerve damage, including potentially irreversible optic nerve damage [9]. In this paper, we present four case reports of patients admitted to the Cardio Thoracic and Vascular Surgery (CTVS) department of a tertiary level healthcare center in North East India, for routine elective procedures, where the patients presented with an interaction following concomitant administration of acenocoumarol and linezolid.

## Case presentation

Case 1

A 72-year-old male patient with type 2 diabetes mellitus and hypertension under medication came for a routine follow-up post-coronary artery bypass graft (CABG) with aortic valve replacement (AVR) for coronary artery disease-double vessel disease (CAD-DVD) with significant aortic stenosis (AS). He had undergone the procedure at the same health facility earlier and had an uneventful and hemodynamically stable stay in the hospital. He was discharged with advice for a follow-up after five days. He was advised to continue acenocoumarol 0.5 mg/1 mg once daily on alternate days and linezolid 600 mg twice daily after food for seven days, along with other medications. On follow-up, vitals were stable, and all test results were within normal limits, with the exception of INR (>10). The patient was immediately admitted and given four units of fresh frozen plasma. Acenocoumarol was withheld, and linezolid was stopped. The patient was discharged when the targeted INR was achieved. Acenocoumarol was restarted post-discharge as the patient had a metallic valve in situ. Upon discharge, the patient was hemodynamically stable and had no further complications.

Case 2

A 64-year-old male patient with a history of hypothyroidism under medication came for a follow-up after five days of discharge, having undergone AVR for aortic stenosis. His stay in the hospital was uneventful. Post-discharge, he was advised to continue acenocoumarol 0.5 mg, one tablet daily, and linezolid 600 mg twice daily for seven days. All his routine test results were within normal limits, but he had a raised INR (>8). He was admitted and administered three units of fresh frozen plasma. INR monitoring was done, and the targeted INR was achieved. While being treated, acenocoumarol was withheld and linezolid was stopped entirely. The patient was discharged and advised to continue acenocoumarol.

Case 3

A 56-year-old female patient came for a follow-up four days post-discharge. She had a history of type 2 diabetes mellitus and hypertension under medication. She had undergone CABG with mitral valve replacement (MVR) for CAD-DVD with severe mitral stenosis at the same healthcare facility, and her stay was uneventful. On discharge, the patient was prescribed acenocoumarol (0.5 mg/1 mg) once daily on alternate days to continue. She was also given linezolid 600 mg twice daily for seven days, along with other medications. All results of routine tests were within normal limits, with the exception of INR (>5). She was given two units of fresh frozen plasma, and the targeted INR was achieved. The patient was hemodynamically stable at discharge, and acenocoumarol was continued as there was a mechanical valve in situ. There were no further complications.

Case 4

A 42-year-old female patient had undergone MVR with a mechanical valve for rheumatic heart disease (RHD) with significant mitral stenosis. She was hemodynamically stable during the entire hospital stay. She was given acenocoumarol 1 mg orally once daily and was also advised to take IV linezolid 600 mg twice daily, among other medications. During the post-operative stay, routine investigations were done, and INR was monitored. On the second post-operative day, the INR was found to be more than 5. The patient was given two units of fresh frozen plasma, and IV linezolid was stopped while acenocoumarol was withheld. The targeted INR was achieved, and the patient was discharged with acenocoumarol and advised for follow-up. She responded well to the treatment and had no further complications during the hospital stay or after discharge.

## Discussion

In the first three cases, the issue of raised INR occurred after discharge during follow-up. However, in the fourth case, the same results were found on the second day of the postoperative stay. In all four cases, the common factor was the concomitant administration of acenocoumarol and linezolid. All other conditions, like comorbidities or other medications, were not common in all four cases, as shown in Tables 1-2.

**Table 1:** Summary of case details. DM: Diabetes mellitus; HTN: Hypertension; CABG: Coronary artery bypass grafting; AVR: Aortic valve replacement; INR: International normalized ratio; MVR: Mitral valve replacement.

Sl. No.	Patient	Comorbidities	Procedure	INR pre treatment	INR post treatment
1	Case 1	Type 2 DM +HTN	CABG+AVR	>10	3.5
2	Case 2	Hypothyroidism	AVR	>8	2.5
3	Case 3	Type 2 DM +HTN	CABG+AVR	>5	2
4	Case 4	None	MVR	>5	2

**Table 2:** Height, weight, and BMI of the patients. BSA: Body surface area; LFT: Liver function test; KFT: Kidney function test.

Case number	Age/Sex	Height (cm)	Weight (kg)	BMI (kg/m^2^)	BSA (m^2^)	LFT		KFT	
						Pre-op	Post-op	Pre-op	Post-op
Case 1	72/M	170	72	24.9	1.83	Within normal limits		Within normal limits	
Case 2	64/M	165	69	25.3	1.76				
Case 3	56/F	163	63	23.7	1.68				
Case 4	42/F	160	65	25.4	1.68				

There were other patients undergoing similar procedures during the same time period. They did not have the concomitant administration of the aforementioned medications or their related complications. This leads us to the conclusion that there is a possible association between the concomitant administration of acenocoumarol and linezolid and raised INR. Acenocoumarol is one of the most commonly used drugs in healthcare in India and is a necessity in the case of patients following cardiac surgeries, while linezolid is one of the most preferred antibiotics used for the prevention of systemic diseases postoperatively. While literature pertaining to various drug interactions of warfarin is present, not much is known about acenocoumarol interactions. One reason for this interaction could be due to the lowering of vitamin K levels by linezolid, due to the decrease of Bifidobacterium, compromising Vitamin K production in the gut [10,11]. One limitation of this case series is the lack of information regarding the plasma levels of acenocoumarol since no such assessment was done.

## Conclusions

Acenocoumarol-Linezolid interaction is a rarely reported, serious but avoidable complication. Further studies are required to understand the mechanisms of this interaction to obtain better clinical insight. If such an interaction goes unnoticed, it can lead to complications in critical care, which may be further accentuated by concomitant drug administration.

## References

[1] Molina CA, Selim MH (2014). Off-label use of new oral anticoagulants: a one-way ticket to nowhere. Stroke.

[2] Head SJ, Bogers AJ, Kappetein AP (2011). New anticoagulants in cardiac surgery. Interv Cardiol Rev.

[3] Minigh J (2008). Acenocoumarol. xPharm, The Comprehensive Pharmacology Reference.

[4] Ansell J, Bergqvist D (2004). Current options in the prevention of thromboembolic disease. Drugs.

[5] Zirlik A, Bode C (2017). Vitamin K antagonists: relative strengths and weaknesses vs. direct oral anticoagulants for stroke prevention in patients with atrial fibrillation. J Thromb Thrombolysis.

[6] Hashemian SM, Farhadi T, Ganjparvar M (2018). Linezolid: a review of its properties, function, and use in critical care. Drug Des Devel Ther.

[7] Eliopoulos GM, Meka VG, Gold HS (2004). Antimicrobial resistance to linezolid. Clin Infect Dis.

[8] (2015). World Health Organization. The selection and use of essential medicines. Twentieth report of the WHO Expert Committee 2015 (including 19th WHO Model List of Essential Medicines and 5th WHO Model List of Essential Medicines for Children). https://www.who.int/publications/i/item/9789241209946.

[9] (2016). Linezolid side effects in detail. https://drugs.com/sfx/linezolid-side-effects.html.

[10] Mishra R, Patel H, Goel B, Vakde T (2019). A case of linezolid toxicity presenting as a sepsis mimic. Case Rep Crit Care.

[11] Sarkar R, Paul R, Kole H (2016). Drug interaction between acenocoumarol and linezolid: case report. JAPI.

